# Clinical Features and Long-Term Outcomes in Very Young Patients with Myocardial Infarction with Non-Obstructive Coronary Arteries

**DOI:** 10.1155/2022/9584527

**Published:** 2022-07-30

**Authors:** Pablo Juan-Salvadores, Víctor Alfonso Jiménez Díaz, Ana Rodríguez González de Araujo, Cristina Iglesia Carreño, Alba Guitián González, Cesar Veiga Garcia, José Antonio Baz Alonso, Francisco Caamaño Isorna, Andrés Iñiguez Romo

**Affiliations:** ^1^Cardiovascular Research Unit, Cardiology Department, Hospital Alvaro Cunqueiro, University Hospital of Vigo, Vigo, Spain; ^2^Cardiovascular Research Group, Galicia Sur Health Research Institute (IIS Galicia Sur), SERGAS-UVIGO, Vigo, Spain; ^3^Interventional Cardiology Unit, Cardiology Department, Hospital Álvaro Cunqueiro, University Hospital of Vigo, Vigo, Spain; ^4^Cardiology Department, Hospital Álvaro Cunqueiro, University Hospital of Vigo, Vigo, Spain; ^5^Department of Preventive Medicine, University of Santiago de Compostela, Santiago de Compostela, Spain; ^6^Consortium for Biomedical Research in Epidemiology and Public Health (CIBER en Epidemiología y Salud Pública-CIBERESP), Santiago de Compostela, Spain

## Abstract

**Background:**

The main cause of acute coronary syndrome (ACS) is coronary artery obstruction due to atherosclerotic plaque growth or thrombus formation secondary to plaque rupture or erosion. However, there is a subgroup of patients with signs and symptoms suggestive of ACS but without relevant coronary artery obstruction on coronary angiography. This population is defined as myocardial infarction with non-obstructive coronary arteries (MINOCA). The present study analyzes the clinical features and outcomes of very young patients with a diagnosis of MINOCA.

**Method:**

Nested case-control study of ≤40-year-old patients referred for coronary angiography due to clinical suspicion of ACS. Patients were divided into three groups: patients with obstructive coronary artery disease (CAD), patients diagnosed with MINOCA, and controls with non-coronary artery disease.

**Results:**

Of 19,321 coronary angiographies performed in our center in a period of 10 years, 408 (2.1%) were in patients ≤40 years old, and MINOCA was identified in 32 (21%) patients. The cardiovascular risk factors for obstructive CAD and MINOCA were very similar. The incidence of major adverse cardiovascular events (MACE) at follow-up was significantly higher in the MINOCA (HR 4.13 (95%CI 1.22–13.89) and obstructive CAD (HR 4.59 (95%CI 1.90–10.99) patients compared to controls. Cocaine use HR 14.58 (95%CI 3.08–69.02), family history of CAD HR 6.20 (95%CI 1.40–27.43), and depression HR 5.16 (95%CI 1.06–25.24) were associated with a poor outcome in the MINOCA population.

**Conclusion:**

Very young patients with MINOCA had a poor prognosis at long-term follow-up, similar to patients with obstructive CAD. Focusing efforts on secondary prevention is essential in this population.

## 1. Introduction

The main cause of acute coronary syndrome (ACS), especially in patients with ST-elevation myocardial infarction (STEMI), is coronary obstruction [1, 2]. However, there is a subgroup of patients who come to the hospital with signs and symptoms suggestive of STEMI without presenting angiographically significant lesions, defined as MINOCA, myocardial infarction with non-obstructive coronary arteries [3]. MINOCA is a heterogeneous disease with multiple pathophysiological pathways [4], and the deepening of its knowledge has led to its inclusion in the Fourth Universal Definition of myocardial infarction to advance knowledge of the etiology, diagnosis, and treatment of MINOCA [4–7].

The prevalence of MINOCA is within 5–25% [8, 9], closer to 6–10% in patients older than 40 years [9, 10]. However, in young patients ≤35 years it is above 18%, which are the most affected population [11]. However, the characteristics of these very young patients have been poorly studied, and some classic risk factors for their clinical manifestation have not been defined. The knowledge available about their long-term prognosis after coronary angiography is much lower. This lack of evidence has generated arbitrariness when selecting the best medical treatment once the diagnosis of MINOCA has been established. All this has a direct impact on patients' health care from now to the future, causing them to become premature chronic coronary artery disease (CAD) patients with a significant economic and health care-needs burden for the community [12]. Hence, focusing efforts on its proper management should be a priority.

The aim of our study is to establish the differences in the characteristics of very young MINOCA patients undergoing coronary angiography compared with patients with typical severe obstructive CAD and with non-coronary artery disease subjects. We also examined these differences in terms of years lived free of major adverse cardiovascular events (MACE) and the risk-adjusted association of the different risk factors.

## 2. Methods

### 2.1. Study Design and Enrolled Population

The trial design entails a nested case-control study in a subset of patients of equal or younger than 40-year-old. All patients had symptoms or clinical suspicion of CAD, including chronic stable angina or ACS, and all underwent their first coronary angiography. The methodology used was published previously [13].

Obstructive CAD (*n* = 250), according to our definition, is described as a significant stenosis in any epicardial coronary artery detected by coronary angiography with a luminal obstruction of ≥75% of the vessel diameter visually assessed or ≥50% by quantitative coronary analysis. Also, the presence of an abnormal resting physiological index was considered as a positive functional significance test of epicardial coronary stenosis. Patients with an obstruction of ≥50% in the left main were considered significant for CAD. MINOCA (*n* = 32) was defined as the presence of myocardial infarction (MI) in the absence of obstructive coronary artery disease (no epicardial major vessel with stenosis ≥50%) and the characteristics described in the latest edition of the Fourth Universal Definition of Myocardial Infarction and the European Society of Cardiology working group position [6, 7]. Subjects with symptoms and clinical suspicions of CAD or with myocardial ischemia documented with a noninvasive test and with non-coronary arteries disease in the coronary angiography, constitute the control group (*n* = 126).

### 2.2. Definition of Variables

All enrolled patients were attended by a tertiary referral center specialized in cardiovascular pathologies. For the management of patients' information, all data was anonymized and disaggregated by means of a specifically developed database for the trial.

All data variables analyzed in the trial were obtained based on the clinical diagnosis or medication used registered on patients' medical records, such as: diabetes mellitus, dyslipidemia, arterial hypertension, body mass index (BMI) defined as obesity >30, smoking (current or former smoker), drug use (patient-recognized or laboratory-tested toxic substances), chronic renal failure (existence of a previous diagnosis or being under chronic hemodialysis treatment), peripheral arterial disease and prior stroke, family history of coronary artery disease (as direct family history of CAD before age 65-year-old for women, and before age 55-year-old for men), atrial fibrillation, depression, and congestive heart failure. The MACE definition included MI, death, new coronary revascularizations, and stroke (the appearance of one of these events for the first time in each patient).

### 2.3. Ethical and Legal Aspects

The Regional Research Ethics Committee approved the conduct of this trial, with registration code 2015/506. All investigators implicated in the study adhered to the applicable ethical and legal standards.

### 2.4. Statistical Analysis

Descriptive statistics are displayed as the mean ± standard deviation (SD) for continuous variables or median with interquartile range (IQR). For all categorical variables, descriptive statistics are shown as numbers and percentages. The statistical analysis plan included a univariate analysis to evaluate differences among the three study groups and their potential statistical significance by means of Fisher's exact test, *χ*^2^ test, Student's *t*-test, or Mann–Whitney *U* test, as appropriate. A multivariate model was developed to establish the contribution of risk combinations using binary logistic regression analysis. Odds ratios (OR) and their 95% confidence intervals were calculated for the cardiovascular risk factors analyzed. Kaplan–Meier curves and log-rank tests were used to compare time to the occurrence of a combination of events among the three groups. In the multivariate Cox regression analysis, the hazard ratio (HR) and 95% confidence intervals were calculated for each combination of events. SPSS 19 for Windows was used to analyze the data.

## 3. Results

From January 1, 2006, to December 31, 2015, a total of 19,321 coronary angiograms were performed in our hospital, and 504 (2.6%) were in patients ≤40 years old. Ninety-six of them were excluded, and 408 (2.1%) patients were ultimately included in the study. Of these, 250 (61.3%) were diagnosed with obstructive CAD, and 152 (37.2%) had nonocclusive lesions, 32 (21.0%) of which had MINOCA, and 120 (79.0%) had non-coronary artery disease (controls).

### 3.1. Features of Study Sample

The female gender was more prevalent in the MINOCA group than in the other 2 study groups. The most frequent symptom described by the patients at their first hospital contact for all groups was chest pain, occurring in 240 (96.0%) of the patients with CAD, 31 (96.9%) of the MINOCA, and 99 (79.8%) of the controls, with statistically significant differences between the MINOCA and the controls (*p* < 0.018). The rest of the baseline characteristics are described in Table 1.

In relation to laboratory parameters, we observed that MINOCA patients presented low-density lipoprotein (LDL) and high-density lipoprotein (HDL) cholesterol levels similar to the patients with obstructive CAD but higher triglyceride values than the controls (182.2 ± 91.0 vs 138.1 ± 83.0; *p*=0.013). The rest of the values are given in Table 2. Among the cardiovascular risk factors studied in the multivariate analysis, only triglyceride levels were related to a higher risk of MINOCA compared to the controls (OR 1.00, 95% CI 1.01–1.01; *p*=0.020). Pharmacological treatment at hospital discharge is displayed in Table 3, showing the great variability found in the management at discharge between the three study groups.

### 3.2. Follow-up

**Figure 1:**
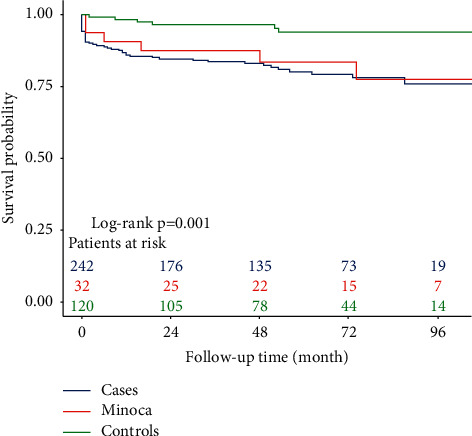
Clinical outcomes of obstructive CAD (cases), MINOCA and control groups at the time of the first MACE.

The mean follow-up time was 6.19 ± 2.4 years in the MINOCA group, 4.98 ± 2.1 years in the obstructive CAD group, and 5.11 ± 2.2 years in the control group. During this time, 69 events were detected, all of them listed in Table 4. MINOCA patients showed a more than 8-fold increase in the risk of suffering new coronary revascularization during follow-up compared to controls (HR 8.43, 95% CI 1.47–48.34; *p*=0.018). Likewise, patients with obstructive CAD presented a more than 6-fold greater risk of suffering new coronary revascularization during follow-up than the controls (HR 6.61, 95% CI 2.28–41.67; *p* < 0.001). At the same time, compared to the control group, MINOCA and obstructive CAD patients showed a higher risk of suffering any of the combined events in the MACE variable (HR 4.13, 95% CI 1.22–13.89, *p*=0.010; and HR 4.59, 95% CI 1.90–10.99, *p* < 0.001; respectively). The poor outcome of MINOCA patients has been associated in the multivariate analysis with cocaine use (HR 14.58, 95% CI 3.08–69.02; *p*=0.010), family history of CAD (HR 6.20, 95% CI 1.40–27.43; *p*=0.016), and depression (HR 5.16, 95% CI 1.06–25.24; *p*=0.043), Table 5. The Kaplan–Meier curves for MACE-free survival of the three study groups are displayed in Figure 1.

## 4. Discussion

To the best of our knowledge, this is the first report describing the long-term outcomes of a very young population of MINOCA patients. In summary, our study revealed the following findings: first, the cardiovascular risk factors are similar to those of patients with obstructive CAD and MINOCA. Second, patients with obstructive CAD and MINOCA patients showed a similar unfavorable long-term prognosis compared to the control group. Third, MINOCA subjects with a history of cocaine use, depression, and a family history of CAD have a higher risk of MACE at long-term follow-up.

The risk profile of MINOCA patients is very similar to those with obstructive CAD. In addition, higher percentages of drug use, depression, and female gender were observed in the MINOCA group. The higher triglyceride levels found in the MINOCA population compared to the other 2 groups were borderline statistically significant but in line with that reported in other studies [14–16]. Also, previous studies have reported lower LDL values for the MINOCA group than for the healthy controls [17] and for obstructive CAD subjects [18]. In our study, the lipid profile and other classic risk factors of MINOCA patients are more similar to those of patients with obstructive CAD than to the control group. These discrepancies may be due to the characteristics of our population, which present the highest values for the different concentrations of lipoproteins in the literature reviewed and the younger population studied.

Hospitalization was comparable to that of patients with obstructive CAD, which may be due to the additional tests performed to confirm the final diagnosis of MINOCA. The pharmacological treatment at hospital discharge varied significantly among the studied groups, highlighting the use of calcium channel blockers in the MINOCA patients compared to the other two groups. The reason may be the prevention of coronary vasospasm and minimizing anginal symptoms. A recent randomized, placebo-controlled trial showed no substantial improvement in coronary vasomotor dysfunction, symptoms, or quality of life with the use of calcium channel blockers, but diltiazem therapy did reduce the prevalence of epicardial spasm [19]. Also, MINOCA subjects received more antiplatelet agents and drugs for dyslipidemia than subjects in the control group, possibly due to their similarity in clinical characteristics with patients with obstructive CAD or due to a clinical perception of a higher risk of subsequent adverse events by their treating physician. However, most of the drugs being used for secondary prevention have been tested in patients with obstructive CAD and are aimed at preventing atherosclerosis. Recent studies in MINOCA patients older than 40 years have shown the benefit on outcomes of prescribing angiotensin-converting enzyme inhibitors or angiotensin II receptor blockers, and their mechanisms of action on the cardiovascular system support these benefits [20]. These medicines are rarely prescribed in our MINOCA population ≤40 years old, which together with statins and beta-blockers are able to improve survival and recurrence of AMI in MINOCA patients >40, finding no benefit for treatment with antiplatelet agents [20, 21]. However, in our study, we did not find any association of statistically significant benefit with any specific pharmacological strategy for the MINOCA patients. It is necessary to carry out randomized clinical trials on this group of patients to help physicians in decision-making regarding the best medical treatment.

During the follow-up, the incidence of MACE in these patients was more than four times higher than in the control group, which was similar to patients with obstructive CAD. Many of the MACE were due to new coronary revascularization. This fact may be due in part to the endothelial dysfunction that is related to an independent predictor of atherosclerosis progression [22, 23]. There is little published data on the long-term outcomes of these very young patients diagnosed with MINOCA. Also, previous studies regarding the prognosis of MINOCA are limited and discordant. The study by Radillis and Pavlakis found a better prognosis for MINOCA patients than for those with obstructive CAD [24]. Nevertheless, a study conducted by Safdar et al. [14] and the data published by Magnani G. et al. [16] confer a similar prognosis to both groups, which is consistent with our study and with a literature review in MINOCA patients older than 40 years [10, 25–27]. Therefore, if these patients present a similar evolution to patients with obstructive CAD, optimal personalized medical treatment should be implemented, together with a closer, standardized clinical follow-up, as in patients with obstructive CAD. Perhaps the use of novel advanced computational techniques such as machine/deep learning could help physicians in the decision-making process for the chronic management of this population [28].

The characteristics of a poor prognosis are different in very young patients with MINOCA than in patients older than 40 years with MINOCA, in which they are related to other risk factors such as hyperglycemia among others [13, 26, 29–31]. The worst prognostic values in follow-up were associated with cocaine use. Its consumption is linked with the appearance of cardiac ischemia for various reasons: it increases oxygen demand, promotes vasoconstriction, and also causes platelet activation, induction of thrombosis, and produces endothelial damage by increasing cell permeability to low-density lipoproteins [32]. Other studies showed a low percentage of cocaine use in young MINOCA [14, 16]. The potential role of a family history of CAD in the development of MINOCA can be attributed to genetic variants and an inherited lifestyle that predispose to CAD [33]. Some studies associate a family history of CAD with high levels of subclinical atherosclerosis, increasing the susceptibility of these subjects to early CAD [34]. Depression can lead to disorders in the normal function of platelet receptors [35], coagulopathic factors such as plasminogen activator inhibitor-1 and fibrinogen, and proinflammatory cytokines [36]. In addition, depression feeds back into classical cardiovascular risk factors such as smoking, inactivity, and obesity [37]. Other authors have described an increased risk of adverse clinical events at follow-up in MINOCA patients with depression. In MINOCA patients with depression other authors found an association with an increased risk of adverse clinical events [38]. Also, they may be affected by a relaxation in secondary prevention measures given the perception by the physician and the patient that it is a benign condition as it does not present significant coronary stenosis.

This study has several limitations. A small proportion of patients had cardiac magnetic resonance and their results were not available in the database. However, the MINOCA diagnostics were performed according to the guidelines and recorded in the hospital discharge report. The presence of nontraditional cardiovascular risk factors, such as interleukin-18, thrombin-activatable fibrinolysis inhibitor, and alteration of PCSK9, was not analyzed. Due to a lack of data in most patients, the association between prothrombotic diseases and ischemic heart disease could not be assessed. As a result of the nature of our study, we cannot rule out selection biases. We include the largest sample size reported in this field of study, which includes a consecutive cohort of patients.

## 5. Conclusion

In our study, very young patients with MINOCA had a poor long-term prognosis, the same as in patients with obstructive CAD. Cocaine use, family history of CAD, and depression increase the risk of MACE at follow-up. Focusing efforts on secondary prevention to prevent recurrences and improve outcomes is necessary in this group of patients.

## Figures and Tables

**Table 1:** Clinical characteristics of patients ≤40 years old undergoing coronary angiography.

Variables	oCAD (*n* = 250)	MINOCA (*n* = 32)	Controls (*n* = 126)	*p* value oCAD vs controls	*p* value MINOCA vs controls	*p* value oCAD vs MINOCA
Age (median and IQR)	37 (34–39)	35 (33–38)	34 (32–39)	0.019	0.862	0.194
Women	28 (11.2%)	7 (21.9%)	17 (13.5%)	0.518	0.238	0.085
Body max index >30	82 (32.8%)	10 (31.3%)	35 (27.8%)	0.321	0.698	0.860
Hypertension	54 (21.6%)	5 (15.6%)	25 (19.8%)	0.693	0.587	0.434
Diabetes	16 (6.4%)	1 (3.1%)	6 (4.8%)	0.523	0.688	0.464
Smoking	213 (85.2%)	24 (75.0%)	78 (61.9%)	<0.001	0.167	0.138
Dyslipidemia	124 (49.6%)	11 (34.4%)	28 (22.2%)	<0.001	0.154	0.105
Family history of CAD	72 (28.8%)	7 (21.9%)	15 (11.9%)	<0.001	0.146	0.411
Illicit drugs and alcohol	56 (22.4%)	9 (28.1%)	26 (20.6%)	0.696	0.362	0.469
Cannabis	30 (12.0%)	4 (12.5%)	8 (6.3%)	0.086	0.241	0.935
Opioids	4 (1.6%)	1 (3.1%)	3 (2.4%)	0.597	0.811	0.538
Alcohol	26 (10.4%)	5 (15.6%)	12 (9.5%)	0.790	0.320	0.374
Cocaine	28 (11.2%)	5 (15.6%)	10 (7.9%)	0.322	0.185	0.463
Peripheral artery disease	3 (1.2%)	0 (0.0%)	1 (0.8%)	0.717	0.613	0.533
Congestive heart failure	1 (0.4%)	0 (0.0%)	1 (0.8%)	0.620	0.613	0.720
Previous stroke	2 (0.8%)	0 (0.0%)	1 (0.8%)	0.995	0.613	0.612
Atrial fibrillation	2 (0.8%)	0 (0.0%)	1 (0.8%)	0.995	0.613	0.612
Renal failure	6 (2.4%)	1 (3.1%)	9 (7.1%)	0.027	0.405	0.804
Depression	16 (6.4%)	5 (15.6%)	11 (8.7%)	0.409	0.248	0.061

Data are given as number (percentage) or mean ± SD. oCAD, obstructive coronary artery disease. CAD, coronary artery disease.

**Table 2:** Laboratory parameters and quantitative variables.

Variables	oCAD (*n* = 250)	MINOCA (*n* = 32)	Controls (*n* = 126)	*p* value oCAD vs controls	*p* value MINOCA vs controls	*p* value oCAD vs MINOCA
Total cholesterol (mg/dl)	203.0 ± 55.4	191.4 ± 35.4	179.6 ± 40.3	<0.001	0.146	0.683
LDL cholesterol (mg/dl)	132.2 ± 51.4	118.3 ± 32.6	110.9 ± 32.3	0.001	0.314	0.431
HDL cholesterol (mg/dl)	38.3 ± 10.4	39.0 ± 10.2	43.4 ± 12.3	0.001	0.085	1.000
Triglycerides (mg/dl)	162.1 ± 109.8	182.2 ± 91.0	138.1 ± 83.0	0.153	0.013	0.904
Creatinine (mg/dl)	1.05 ± 1.1	0.92 ± 0.2	1.54 ± 2.4	0.027	0.010	1.000
Glucose (mg/dl)	107.4 ± 41.4	101.6 ± 23.4	102.3 ± 45.9	0.892	0.935	1.000
LVEF (%)	54.4 ± 8.8	58.9 ± 8.7	56.7 ± 10.8	0.378	0.320	0.111
Hospitalization days	7.3 ± 7.4	7.3 ± 6.6	5.1 ± 8.5	0.038	0.476	1.000

Data are given as mean ± SD. oCAD, obstructive coronary artery disease. CAD, coronary artery disease. HDL, high-density lipoproteins. LDL, low-density lipoproteins. LVEF, left ventricle ejection fraction.

**Table 3:** Pharmacological treatment at hospital discharge.

Pharmacological treatment	Hospital discharge					
	oCAD (*n* = 250)	MINOCA (*n* = 32)	Controls (*n* = 126)	*p* value oCAD vs controls	*p* value MINOCA vs controls	*p* value oCAD vs MINOCA
P2Y12 Inhibitors	229 (94.7%)	13 (41.9%)	16 (13.4%)	<0.001	<0.001	<0.001
ASA	243 (100%)	25 (80.6%)	36 (30.3%)	<0.001	<0.001	<0.001
Anticoagulants	9 (3.8%)	0 (0.0%)	4 (3.4%)	0.853	0.305	0.277
Beta-blockers	200 (84.7%)	11 (35.5%)	23 (19.5%)	<0.001	0.059	<0.001
ACE inhibitors	117 (49.4%)	1 (3.2%)	12 (10.2%)	<0.001	0.223	<0.001
ARBs	10 (4.2%)	0 (0.0%)	10 (8.5%)	0.104	0.093	0.243
Calcium channel blockers	13 (5.5%)	10 (32.3%)	9 (7.6%)	0.436	<0.001	<0.001
Statins	221 (92.9%)	17 (54.8%)	18 (15.4%)	<0.001	<0.001	<0.001
Diuretics	17 (7.2%)	0 (0.0%)	8 (6.8%)	0.883	0.143	0.129
Antidiabetics	2 (0.9%)	1 (3.2%)	0 (0.0%)	0.315	0.050	0.239
Antiarrhythmics	5 (2.1%)	0 (0.0%)	3 (2.5%)	0.800	0.370	0.413

Data are given as number (percentage). oCAD, obstructive CAD. ASA, acetylsalicylic acid. ACE inhibitors; angiotensin-converting enzyme inhibitors. ARBs, angiotensin II receptor blocker.

**Table 4:** Adverse events in the study period.

Adverse events	oCAD (*n* = 243)	MINOCA (*n* = 32)	Controls (*n* = 120)	*p* value oCAD vs controls	*p* value MINOCA vs controls	*p* value oCAD vs MINOCA
New coronary revascularizations	34 (14.0%)	4 (12.0%)	1 (1.7%)	<0.001	0.018	1.000
Death	8 (3.3%)	2 (6.2%)	4 (3.3%)	1.000	0.607	0.330
AMI	8 (3.3%)	1 (3.1%)	1 (0.8%)	0.282	0.378	1.000
Stroke	5 (2.1%)	0 (0.0%)	1 (0.8%)	0.668	1.000	1.000
MACE	47 (19.3%)	6 (18.8%)	6 (5.0%)	<0.001	0.020	0.928

Data are given as number (percentage). oCAD, obstructive CAD. AMI, acute myocardial infarction. MACE, major adverse cardiovascular events, a composite of death, myocardial infarction, stroke, and new coronary revascularizations.

**Table 5:** Risk factors associated with MACE.

MACE	Hazard ratio 95%CI	*p* value
BMI>30	0.19 (0.26 – 1.43)	0.108
Hypertension	8.26 (0.96 – 30.94)	0.054
Diabetes mellitus	1.74 (0.04 – 31.69)	0.770
Smoking	4.98 (0.61 – 20.33)	0.132
Dyslipidemia	0.47 (0.63 – 3.64)	0.477
Family history of CAD	6.20 (1.40 – 27.43)	0.016
Depression	5.16 (1.06 – 25.24)	0.043
Cocaine	14.58 (3.08 – 69.02)	0.010

BMI, body max index. CAD, coronary artery disease. CI, confidence Interval. MACE, major adverse cardiovascular events, a composite of death, myocardial infarction, stroke, and new coronary revascularizations.

## Data Availability

The data supporting the results in the current study are available from the corresponding author upon reasonable request.
